# Supplementary material for: upsML: A high-accuracy machine learning classifier for predicting Plasmodium falciparum var gene upstream groups
Source: PLoS One. 2026 Apr 16;21(4):e0344557. doi: 10.1371/journal.pone.0344557 (PMC13086428; doi:10.1371/journal.pone.0344557)
Supplement: S1 Table — (PDF) [file pone.0344557.s001.pdf]

S1 Table: Complete Training Set of Full *Var* Genes and ups Assignments used.

			based on Rask2010 phylo			Supp
Sample	Study	**Var (NTS)**	upsA	upsB	upsC	upsE
3D7	Reference	61	10	37	14	0
IGH	Rask 2010	59	14	35	10	1
RAJ116		53	10	30	13	1
GN01	Otto 2019	80	17	52	11	0
CD01		70	12	44	14	0
Dd2		46	11	25	10	1
KE01		51	7	27	17	0
KH01		58	10	29	19	0
GA01		58	7	40	11	0
GB4		66	19	36	11	0
KH02		49	7	29	13	0
IT		55	10	32	13	0
SD01		49	8	27	14	0
TG01		124	26	78	20	0
HB3		44	8	26	10	2
SN01		67	15	39	13	0
ML01		104	16	61	27	0
7G8	Moser 2020	41	6	25	10	0
NF166.C8		60	12	39	9	0
NF135.C10		54	10	31	13	0
CO01	Ruiz 2023	47	5	29	13	0
KE07		49	14	20	15	0
2004		67	17	39	11	0
Gambia var genes	Classens unpublished	2835	519	1752	564	105
	**TOTAL**	4247	790	2582	875	110
