# Supplementary material for: upsML: A high-accuracy machine learning classifier for predicting Plasmodium falciparum var gene upstream groups
Source: PLoS One. 2026 Apr 16;21(4):e0344557. doi: 10.1371/journal.pone.0344557 (PMC13086428; doi:10.1371/journal.pone.0344557)
Supplement: S2 Table — (PDF) [file pone.0344557.s002.pdf]

S2 Table: All and Unique Training Sets of Isolated Var Genes Components Regions

	Isolated Gene Regions				After Filtering for Similar Sub-Sequence			
Sample	***Pf*EMP1**	**'Exon 1'**	**Cassette**	**Tag**	***Pf*EMP1**	**'Exon 1'**	**Cassette**	**Tag**
IGH	60	57	54	58	60	57	53	57
RAJ116	54	49	45	50	54	49	41	45
GN01	80	76	75	80	79	76	68	72
CD01	70	68	64	67	68	66	63	63
Dd2	47	46	41	44	44	43	39	43
KE01	51	50	50	50	47	47	44	43
KH01	58	56	53	57	56	55	46	48
GA01	58	55	54	58	56	54	47	47
GB4	66	64	57	62	60	58	50	50
KH02	49	48	44	47	47	46	38	40
IT	55	55	49	54	49	49	40	41
SD01	49	49	46	46	49	49	40	37
TG01	124	122	108	120	122	120	98	105
HB3	46	44	39	43	46	43	37	40
SN01	67	67	59	66	65	63	54	57
ML01	104	93	97	99	104	93	94	90
7G8	41	41	39	40	41	41	36	33
NF166.C8	60	60	56	56	59	58	50	50
NF135.C10	54	54	51	51	50	50	44	39
CO01	47	46	45	46	44	43	39	35
KE07	49	44	41	47	45	39	34	36
2004	67	67	58	63	65	65	54	58
Gambia var genes	2940	2741	2591	2752	1220	1115	886	841
**TOTAL**	4296	4052	3816	4056	2530	2379	1995	1970
