# Supplementary material for: upsML: A high-accuracy machine learning classifier for predicting Plasmodium falciparum var gene upstream groups
Source: PLoS One. 2026 Apr 16;21(4):e0344557. doi: 10.1371/journal.pone.0344557 (PMC13086428; doi:10.1371/journal.pone.0344557)
Supplement: S3 Table — (PDF) [file pone.0344557.s003.pdf]

S3 Table: Overall accuracy of upsML, including shorter peptide models

		Overall Accuracy					
		TRAIN/TEST 80/20					
		**Linear**	**Poly**	**RBF**	**Sigmoid**	**RanFor**	**XGB**
**tag**	**Amino Acid**	0.77	0.75	0.76	0.77	0.77	0.77
**cassette**		0.76	0.76	0.77	0.75	0.76	0.77
**'exon 1'**		0.73	0.70	0.75	0.70	0.74	0.75
***Pf*EMP1**		0.76	0.75	0.79	0.76	0.77	0.79
**tag**	**Dipeptide**	0.78	0.81	0.82	0.78	0.81	0.81
**cassette**		0.80	0.79	0.81	0.79	0.80	0.83
**'exon 1'**		0.82	0.81	0.83	0.70	0.79	0.81
***Pf*EMP1**		0.85	0.85	0.86	0.73	0.83	0.85
**tag**	**Tetrapeptide**	0.79	0.82	0.81	0.75	0.80	0.79
**cassette**		0.85	0.83	0.84	0.84	0.81	0.85
**'exon 1'**		0.87	0.85	0.86	0.86	0.85	0.87
***Pf*EMP1**		0.91	0.87	0.90	0.91	0.85	0.90
**tag**	**Tetrapeptide (unassigned as B)**	0.83	0.85	0.85	0.84	0.84	0.84
**cassette**		0.87	0.86	0.86	0.86	0.83	0.86
**'exon 1'**		0.89	0.88	0.89	0.90	0.88	0.90
***Pf*EMP1**		0.92	0.89	0.92	0.92	0.87	0.90
