# Supplementary material for: upsML: A high-accuracy machine learning classifier for predicting Plasmodium falciparum var gene upstream groups
Source: PLoS One. 2026 Apr 16;21(4):e0344557. doi: 10.1371/journal.pone.0344557 (PMC13086428; doi:10.1371/journal.pone.0344557)
Supplement: S4 Table — (PDF) [file pone.0344557.s004.pdf]

S4 Table. upsML Model Speeds when training then classifying 60 3D7 complete *Pf*EMP1 sequences

		Times (hh:mm:ss)
*Pf*EMP1	SVM linear (tetra)	0:00:10
	SVM poly (tetra)	0:19:17
	SVM rbf (tetra)	0:15:01
	XGBoost (tetra)	0:29:00
