# Supplementary material for: upsML: A high-accuracy machine learning classifier for predicting Plasmodium falciparum var gene upstream groups
Source: PLoS One. 2026 Apr 16;21(4):e0344557. doi: 10.1371/journal.pone.0344557 (PMC13086428; doi:10.1371/journal.pone.0344557)
Supplement: S5 Table — Sensitivity refers to the proportion of true positives, while specificity refers to the proportion of true negatives. (PDF) [file pone.0344557.s005.pdf]

**S5 Table. Specificity and Sensitivity of Tetrapeptide Models for first model.** Sensitivity refers to the proportion of true positives, while specificity refers to the proportion of true negatives.

| | | **TAG TETRAPEPTIDE** | | | | | |
|---|---|---|---|---|---|---|---|
| | | **Linear** | **Poly** | **RBF** | **Sigmoid** | **RanFor** | **XGBoost** |
| **A (n=67)** | **Sensitivity** | 1.000 | 1.000 | 1.000 | 1.000 | 0.970 | 1.000 |
| | **Specificity** | 0.997 | 0.997 | 0.997 | 0.997 | 0.997 | 0.991 |
| **B (n=261)** | **Sensitivity** | 0.939 | 0.950 | 0.962 | 0.962 | 0.992 | 0.923 |
| | **Specificity** | 0.624 | 0.654 | 0.632 | 0.602 | 0.526 | 0.662 |
| **C (n=66)** | **Sensitivity** | 0.242 | 0.303 | 0.258 | 0.197 | 0.076 | 0.318 |
| | **Specificity** | 0.954 | 0.963 | 0.973 | 0.973 | 0.997 | 0.948 |
| | | **CASSETTE TETRAPEPTIDE** | | | | | |
| | | **Linear** | **Poly** | **RBF** | **Sigmoid** | **RanFor** | **XGBoost** |
| **A (n=47)** | **Sensitivity** | 1.000 | 1.000 | 1.000 | 1.000 | 1.000 | 1.000 |
| | **Specificity** | 1.000 | 1.000 | 1.000 | 1.000 | 1.000 | 1.000 |
| **B (n=265)** | **Sensitivity** | 0.970 | 0.974 | 0.955 | 0.958 | 0.981 | 0.962 |
| | **Specificity** | 0.672 | 0.627 | 0.687 | 0.664 | 0.537 | 0.672 |
| **C (n=87)** | **Sensitivity** | 0.494 | 0.425 | 0.517 | 0.483 | 0.287 | 0.494 |
| | **Specificity** | 0.974 | 0.978 | 0.962 | 0.965 | 0.984 | 0.968 |
| | | **'EXON 1' TETRAPEPTIDE** | | | | | |
| | | **Linear** | **Poly** | **RBF** | **Sigmoid** | **RanFor** | **XGBoost** |
| **A (n=73)** | **Sensitivity** | 1.000 | 0.973 | 1.000 | 1.000 | 1.000 | 1.000 |
| | **Specificity** | 1.000 | 1.000 | 1.000 | 1.000 | 1.000 | 1.000 |
| **B (n=298)** | **Sensitivity** | 0.956 | 0.980 | 0.956 | 0.956 | 0.993 | 0.970 |
| | **Specificity** | 0.792 | 0.702 | 0.781 | 0.798 | 0.680 | 0.775 |
| **C (n=100)** | **Sensitivity** | 0.630 | 0.490 | 0.610 | 0.640 | 0.430 | 0.600 |
| | **Specificity** | 0.965 | 0.984 | 0.965 | 0.965 | 0.995 | 0.976 |
| **E (n=5)** | **Sensitivity** | 1.000 | 1.000 | 1.000 | 1.000 | 1.000 | 1.000 |
| | **Specificity** | 1.000 | 1.000 | 1.000 | 1.000 | 1.000 | 1.000 |
| | | ***Pf*EMP1 TETRAPEPTIDE** | | | | | |
| | | **Linear** | **Poly** | **RBF** | **Sigmoid** | **RanFor** | **XGBoost** |
| **A (n=98)** | **Sensitivity** | 1.000 | 0.969 | 1.000 | 1.000 | 0.990 | 1.000 |
| | **Specificity** | 1.000 | 0.998 | 0.998 | 1.000 | 0.993 | 1.000 |
| **B (n=296)** | **Sensitivity** | 0.939 | 0.936 | 0.946 | 0.949 | 0.926 | 0.943 |
| | **Specificity** | 0.890 | 0.829 | 0.886 | 0.886 | 0.781 | 0.848 |
| **C (n=101)** | **Sensitivity** | 0.772 | 0.673 | 0.762 | 0.762 | 0.554 | 0.683 |
| | **Specificity** | 0.956 | 0.956 | 0.963 | 0.963 | 0.953 | 0.958 |
| **E (n=11)** | **Sensitivity** | 1.000 | 1.000 | 1.000 | 1.000 | 1.000 | 1.000 |
| | **Specificity** | 1.000 | 1.000 | 1.000 | 1.000 | 1.000 | 1.000 |
