# Supplementary material for: upsML: A high-accuracy machine learning classifier for predicting Plasmodium falciparum var gene upstream groups
Source: PLoS One. 2026 Apr 16;21(4):e0344557. doi: 10.1371/journal.pone.0344557 (PMC13086428; doi:10.1371/journal.pone.0344557)
Supplement: S6 Table — (PDF) [file pone.0344557.s006.pdf]

S6 Table. Confusion Matrixes of Internal / Subtelomeric Models.

		A				B_sub				CB_int				E			
		n=67				n=135				n=108				N/A			
		A	B_sub	CB_int	E	A	B_sub	CB_int	E	A	B_sub	CB_int	E	A	B_sub	CB_int	E
TAG	SVM linear (tetra)	67				1	98	36			36	72					
	SVM poly (tetra)	66	1				112	23			45	63					
	SVM rbf (tetra)	67					103	32			37	71					
	SVM sigmoid (tetra)	67				1	99	35			38	70					
	RanFor (tetra)	66	1				110	25			43	65					
	XGBoost (tetra)	66	1			2	98	35		1	42	65					
		n=44				n=129				n=126				N/A			
		A	B_sub	CB_int	E	A	B_sub	CB_int	E	A	B_sub	CB_int	E	A	B_sub	CB_int	E
CASSETTE	SVM linear (tetra)	44					105	24			35	101					
	SVM poly (tetra)	44					106	23			39	97					
	SVM rbf (tetra)	44					109	20			36	100					
	SVM sigmoid (tetra)	44					108	21			38	98					
	RanFor (tetra)	44					105	24			35	101					
	XGBoost (tetra)	44					104	25			38	98					
		n=85				n=136				n=148				n=9			
		A	B_sub	CB_int	E	A	B_sub	CB_int	E	A	B_sub	CB_int	E	A	B_sub	CB_int	E
'EXON 1'	SVM linear (tetra)	85					113	23			38	110					9
	SVM poly (tetra)	85					114	22			43	105					9
	SVM rbf (tetra)	85					115	21			40	108					9
	SVM sigmoid (tetra)	85					110	26			39	109					9
	RanFor (tetra)	85					116	20			48	100					9
	XGBoost (tetra)	85					114	22			39	109					9
		n=78				n=156				n=153				n=9			
		A	B_sub	CB_int	E	A	B_sub	CB_int	E	A	B_sub	CB_int	E	A	B_sub	CB_int	E
Pf EMP1	SVM linear (tetra)	78				2	145	9			22	131					9
	SVM poly (tetra)	77	1			2	143	11			19	134					9
	SVM rbf (tetra)	78				2	144	10			21	132					9
	SVM sigmoid (tetra)	78				1	144	11			20	133					9
	RanFor (tetra)	77	1			5	139	12			26	127					9
	XGBoost (tetra)	78					145	11			20	133					9
