# Supplementary material for: upsML: A high-accuracy machine learning classifier for predicting Plasmodium falciparum var gene upstream groups
Source: PLoS One. 2026 Apr 16;21(4):e0344557. doi: 10.1371/journal.pone.0344557 (PMC13086428; doi:10.1371/journal.pone.0344557)
Supplement: S7 Table — Split of results for the different sequence types. (PDF) [file pone.0344557.s007.pdf]

S7 Table. Sensitivity and Specificity of Internal/Subtelomeric Models. Split of results for the different sequence types.

		Linear	Poly	RBF	Sigmoid	RanFor	XGBoost
		**TAG TETRAPEPTIDE**					
**A (n=67)**	**Sensitivity**	1.000	0.985	1.000	1.000	0.985	0.985
	**Specificity**	0.996	1.000	1.000	0.996	1.000	0.988
**B_sub (n=135)**	**Sensitivity**	0.726	0.830	0.763	0.733	0.815	0.726
	**Specificity**	0.794	0.737	0.789	0.783	0.749	0.754
**BC_int (n=108)**	**Sensitivity**	0.667	0.583	0.657	0.648	0.602	0.602
	**Specificity**	0.822	0.886	0.842	0.827	0.876	0.827
		**CASSETTE TETRAPEPTIDE**					
		**Linear**	**Poly**	**RBF**	**Sigmoid**	**RanFor**	**XGBoost**
**A (n=44)**	**Sensitivity**	1.000	1.000	1.000	1.000	1.000	1.000
	**Specificity**	1.000	1.000	1.000	1.000	1.000	1.000
**B_sub (n=129)**	**Sensitivity**	0.814	0.822	0.845	0.837	0.814	0.806
	**Specificity**	0.806	0.783	0.800	0.789	0.806	0.789
**BC_int (n=136)**	**Sensitivity**	0.743	0.713	0.735	0.721	0.743	0.721
	**Specificity**	0.861	0.867	0.884	0.879	0.861	0.855
		**'EXON 1' TETRAPEPTIDE**					
		**Linear**	**Poly**	**RBF**	**Sigmoid**	**RanFor**	**XGBoost**
**A (n=85)**	**Sensitivity**	1.000	1.000	1.000	1.000	1.000	1.000
	**Specificity**	1.000	1.000	1.000	1.000	1.000	1.000
**B_sub (n=136)**	**Sensitivity**	0.831	0.838	0.846	0.809	0.853	0.838
	**Specificity**	0.843	0.822	0.835	0.839	0.802	0.839
**BC_int (n=148)**	**Sensitivity**	0.743	0.709	0.730	0.736	0.676	0.736
	**Specificity**	0.900	0.904	0.909	0.887	0.913	0.904
**E (n=9)**	**Sensitivity**	1.000	1.000	1.000	1.000	1.000	1.000
	**Specificity**	1.000	1.000	1.000	1.000	1.000	1.000
		***Pf*EMP1 TETRAPEPTIDE**					
		**Linear**	**Poly**	**RBF**	**Sigmoid**	**RanFor**	**XGBoost**
**A (n=78)**	**Sensitivity**	1.000	0.987	1.000	1.000	0.987	1.000
	**Specificity**	0.994	0.994	0.994	0.997	0.984	1.000
**B_sub (n=156)**	**Sensitivity**	0.929	0.917	0.923	0.923	0.891	0.929
	**Specificity**	0.908	0.917	0.913	0.917	0.888	0.917
**BC_int (n=153)**	**Sensitivity**	0.856	0.876	0.863	0.869	0.830	0.869
	**Specificity**	0.963	0.955	0.959	0.955	0.951	0.955
**E (n=9)**	**Sensitivity**	1.000	1.000	1.000	1.000	1.000	1.000
	**Specificity**	1.000	1.000	1.000	1.000	1.000	1.000
