# Supplementary material for: upsML: A high-accuracy machine learning classifier for predicting Plasmodium falciparum var gene upstream groups
Source: PLoS One. 2026 Apr 16;21(4):e0344557. doi: 10.1371/journal.pone.0344557 (PMC13086428; doi:10.1371/journal.pone.0344557)
Supplement: S1 Fig — Data from Table 6, of 660 field isolates, from 11 regions, classified with upsML. The genes were grouped as follows: Asia: Thailand, Cambodia, Vietnam and Laos (median 32.6); East Africa: Mali, Kenya, Malawi & Congo (median 28.1); West Africa: Senegal, The Gambia, Mali and Ghana (median 28.8). Isolates with fewer than 30 var genes in the assembly were ignored. The differences in abundance between Asia and the two African regions were significant, as indicated by a Welch Two-Sample t-test (both <1e-08). (PDF) [file pone.0344557.s008.pdf]

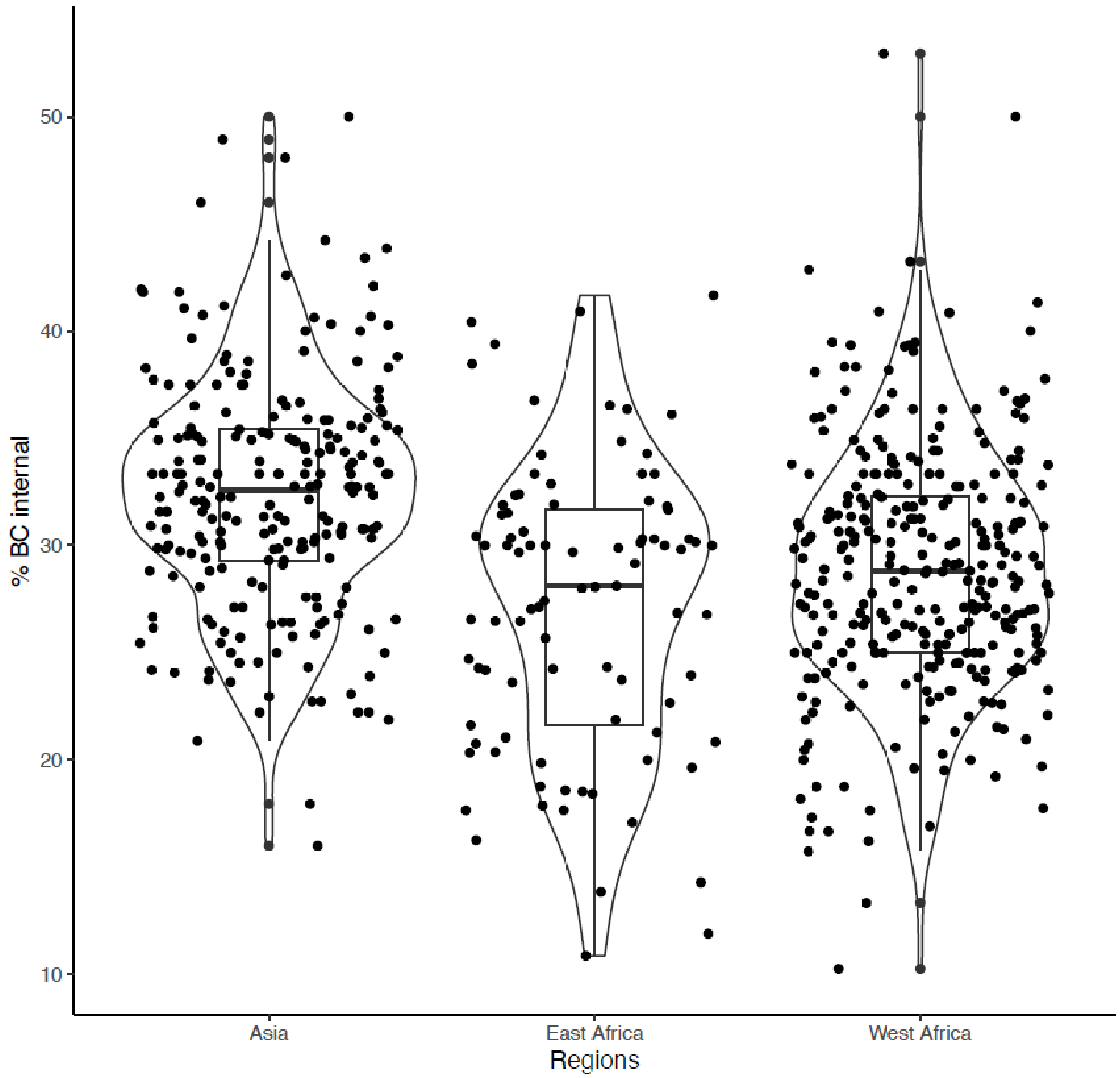

S1 Fig. Relative number of internal *var* genes per region. Data from Table 6, of 660 field isolates, from 11 regions, classified with upsML. The genes were grouped as follows: Asia: Thailand, Cambodia, Vietnam and Laos (median 32.6); East Africa: Mali, Kenya, Malawi & Congo (median 28.1); West Africa: Senegal, The Gambia, Mali and Ghana (median 28.8). Isolates with fewer than 30 *var* genes in the assembly were ignored. The differences in abundance between Asia and the two African regions were significant, as indicated by a Welch Two-Sample t-test (both < 1e-08).
