# Supplementary material for: upsML: A high-accuracy machine learning classifier for predicting Plasmodium falciparum var gene upstream groups
Source: PLoS One. 2026 Apr 16;21(4):e0344557. doi: 10.1371/journal.pone.0344557 (PMC13086428; doi:10.1371/journal.pone.0344557)
Supplement: Attachment — Submitted filename: plosone25.pdf [file pone.0344557.s009.pdf]

Review of "upsAI: A high-accuracy machine learning classifier for predicting *Plasmodium falciparum var* gene upstream groups"

In this paper, the authors develop a new machine learning method (based on support vector machines) to classify the upstream sequences of the *var* genes of *Plasmodium falciparum*, based on the protein sequences (or subsequences) of the gene itself. They show that their method is able to successfully classify with high accuracy, including on the upsB/C divide which has previously been difficult to separate, and outperforms current methods in the literature.

The paper is well-written and easy to read, and the problem it tackles is interesting and topical. The results are good, and represent a significant advance on the current state-of-the-art. There are some issues that I would like to see addressed, and areas that I would like to see explored a bit further, but overall I think it is a nice addition to the literature.

Major comments:

1. The characterisation of the method as an "AI" method is inaccurate. The methods used here are well-known and well-established machine learning methods, but they are not AI methods in the modern sense of deep learning/neural networks. Having the term "AI" in the title and in the name of the method is misleading at best; when I read the title, I was indeed expecting some kind of deep neural network. To further characterise the method as "explainable AI", when this is unrelated to the modern (and topical) sense of extracting meaning from "black box"-style models, seems to me to be a little disingenuous.

2. Why were the models only trained with 1-, 2-, and 4-mers? The improving performance with respect to length suggests that longer lengths should have been tried. I understand that at some point there are too many feature dimensions, but certainly 3-mers (at the very least) should have been tried.

3. It is somewhat improper to attempt several different machine learning methods and report the best classification accuracy among them as the final numerical answer. Due to random variation, one of the methods will always be slightly better than the others, but not systematically. Indeed, this is exactly what we see in (e.g.) Table

1, where there is (to my eye) no significant difference between the methods, but the best number among them is being claimed as the "overall accuracy". This leads to different methods being used for different input data types, for no biological reason. What should be done is that a single method should be chosen based on this Table (probably the linear SVM), and the accuracy of that one method reported from then on.

4. l289: I would not characterise this as a "classifier for genome location"; I would expect such a thing to predict the actual position in the genome. What is being done here is rearranging the upsB/C divide according to genome location. I think this is interesting enough in its own right without overselling it. Likewise, I would consider l325–326 as somewhat overselling the ability to predict chromosomal position.

5. Figure 4: In general I like this idea a lot and I think it clearly shows the inclination of various subsequences towards the different ups types, which to my knowledge has not been studied before. However, the details of the method used here need to be expanded; the Methods section (l539) does not give the mathematical formulas used. I can guess what is going on, but I should not have to. In the Results section, the AUC score is not defined in l339. I was hoping that it would be defined later, but when I read the relevant Methods section, there does not appear to be any mention of AUC.

   In fact, I think more could be done in this direction. The particular sequence studied shows a tendency towards upsB in the tag region but upsC in other parts of the PfEMP1 sequence. In the discussion this is considered as a signal of recombination. There is already a well-known method (Zilversmit *et al.*, cited in the paper) for detecting recombination in *var* genes; why not use it here to determine that there has indeed been a recombination in this particular sequence, and what the parents were? That would lend considerably more weight to this analysis.

   When a linear projection is used for classification, the "loading" weights (the coefficients of the projection matrix) are usually used to explain the relative importance of the various features. In this case, this seems like an obvious thing to do, and would ascribe importance to the various 4-mers which could be then investigated for biological meaning. Was this considered?

   Finally, the claim (l419) that the drops are aligned with recombination breakpoints should be substantiated, by (e.g.) comparison to homology block (HB) locations.

6. l379: There should be more analysis of the results here than a simple table. The text considers that it is "informative to observe geographical variation", but what information should be observed here?

7. l506: I am somewhat disturbed that using 1-mers can achieve any classification value at all. This is equivalent to using (single) amino acid frequencies to classify the sequences, analogous to using GC-content for DNA sequences. There should be very

little discriminatory value there, but instead the performance (for e.g., tags) is only slightly worse than the full method. Why is this so?

More generally, the method does not use any (sequence) positional information other than local (sub-)sequences in the form of k-mers. Again, why is this? Is position unnecessary for classification?

8. There is somewhat of an overall lack of mathematical detail in the description of the methods. I am reasonably familiar with the methods used, but the manuscript should be self-contained. Also, it appears that the methods were used "out-of-the-box" without specific adaptation to the task at hand. This is okay, but perhaps not ideal.

Minor comments:

- In general, there should be a space before citations, e.g., l51: "to the iRBC surface (1)".
- l22: "and 92% for"
- l75: "var2csa" (capitalisation)
- l84: Refer to Figure 1 in the text.
- l85: I suggest using parentheses, e.g., "subtelomeric regions (upsA/B/E)" as the current punctuation is unclear.
- l138: This seems to imply that 110 out of the 172 genes were identified as upsE, which seems absurd — I suggest rewording.
- Figure 2: To better understand the reliability of the annotations, the annotations mapped from the Rask dataset should be highlighted in this figure, or better yet shown by themselves in a companion figure.
- l162: To exclude a particular genome for validation risks that the genes included there are not a random sample; I think this is probably okay in this instance since this genome is not used to assess accuracy.
- l167: "with tetrapeptides, and we did"
- l185: incomplete sentence
- Table 4: extra M in "HMM"
- l252: last sentence is ungrammatical

- l256–260: The presentation of numbers here is hard to read. A table would be better, and confusion matrices for each method would be even better.
- l272: *Pf3D7_0461300* is the only upsC sequence, there is another upsB sequence.
- l366: the "varDB" dataset has not been named before; I presume it is the dataset that is being studied here, but that should be made clear.
- l403: The wording here is awkward: evaluating multiple classifiers is not the non-trivial task.
- l409: I was unable to find the corresponding table entry for the 0.27 figure. Also, it actually undersells the method to cite the lowest figure, so I suggest revision.
- l413: Although it is good to see a biological explanation being advanced, I do not find this one very plausible; exon 1 lies between the ups region and exon 2, so it would be difficult (albeit not impossible) to have more linkage between ups and exon 2.
- l425: Although this is likely true, this ceiling does not appear to exist for upsA/E genes, which are classified almost perfectly.
- l435: Again, this is likely true. However, the nature of the cUps method suggests that this effect is probably small, since it is a HMM-based method which does not classify to the nearest neighbour in the dataset. In contrast, SVM-based methods, although far more sophisticated, do tend to classify to the nearest neighbour, since the individual sample points do determine the classification boundary.
- l473: I believe modern ML-based methods (e.g., RAxML, IQ-TREE) would be able to accommodate datasets of this size, and are more accurate than FastTree. Were they tried?
- l491: capitalisation
- l495, 552: "Python" (capitalisation)
- l512: "*var* gene"
- l512: The formulas for the various metrics should be stated here.
- l513: "sensitivity"
- l531: "the remaining genes" seems to imply the existence of non-*var* genes in the dataset, which is almost certainly not what is meant.
- l531: How were the positions verified?
- l538: The datasets here are smaller than before — why is this?

- l546: I am a little confused about the point of this entire section — is this part of the dataset used for training, described earlier? If so, why describe it again, and if not, why mention it at all?
