# Supplementary material for: upsML: A high-accuracy machine learning classifier for predicting Plasmodium falciparum var gene upstream groups
Source: PLoS One. 2026 Apr 16;21(4):e0344557. doi: 10.1371/journal.pone.0344557 (PMC13086428; doi:10.1371/journal.pone.0344557)
Supplement: Attachment — Submitted filename: upsML.responce2editor.Rebuttal.pdf [file pone.0344557.s010.pdf]

Dear Editor,

Thank you for the constructive and positive evaluation of our manuscript. We have carefully addressed all reviewer comments and clarified the points raised. While we acknowledge the reviewers' suggestions for additional analyses, we believe that such extensive investigations fall outside the current scope of our study. Our primary aim is to demonstrate the potential of our tool *upsML* rather than to provide a full in-depth analysis of all possible applications.

We hope that our revisions and clarifications adequately address the reviewers' concerns and that the manuscript is now suitable for publication.

We also included all the formatting requests.

Best regards,
Thomas Otto

Reviewer #1:

Pangilinan et al present a new tool for predicting the upstream region classification of var genes encoding P .falciparum's key virulence proteins , PfEMP1). This is important as the var genes, despite their extreme diversity generated though both recombination and mutation, are well organized in the genome with specific receptor-binding phenotypes linked to specific chromosomal locations and upstream regions. This has led reserachers to use these sequence signatures to link receptor binding phenotypes to clinical outcome, and speculate if also genetic regulation may be tied to e.g. parasites establishing low patent chronic "dry season "infections or fast switching at the eary onset of infection etc. The present tool for linking ups-type to gene fragments which can be extracted experimentally- most commonly and DBLa tags is a welcoming tool, not least as an earlier attempt for such a tool was considered highly inaccurate – a notion supported by the present analyses.

A main challenge is within the biological limitations provided by the recombinogenic nature of the var genes.. ie the "truth" is that most likely not all var genes can be classified as either upsa, b or c – due to their recent recombination between the groups. As In addition to the upsABC the authors attempt to predict the chromosomal location from encoded sequence fragments, - again this may be inherently difficult for recently recombined genes.
On this background the authors apply and compare three different computational approaches to tackle the classification. The reporting on this is sound hones and open about the limitations – which - as expected and supported by 2 examples of in-depth analysis of ups-type calling sequence elements tiled across the var sequence, - appear to reflect said biological limitations.
It is this open and clear description of the limitations of the too, which makes the strength of the paper and will guide proper applications and data interpretations from the tool. I think this is well done.

Answer:

We would like to thank the reviewer for their throughtful and positive reply.

A key application will be the annotation of expressed DBLa tags as these are the most easily acquired through NGS. A challenge for using the tool is that it relies on protein sequence. Have the authors tried to apply raw error prone DBLa tag DNA sequences ? if this was possible this would omit need for cumbersome data curation of large DBLa tag datasets?

Answer:

We appreciate this important suggestion. At present, *upsML* requires error-free translated protein sequences as input. Our tests with raw, error-prone DBLa tag DNA sequences confirmed that *upsML* does not perform reliably without prior error correction or translation.

We agree that developing methods to handle raw sequences directly would greatly simplify the workflow. Indeed, we are exploring a separate pre-processing pipeline to translate and

error-correct DBLa tags prior to annotation, but we view this as a complementary tool rather than a core component of *upsML*.

In my view the use of DC is less likely to happen or be meaningful. The mention of domain cassettes (DCs) line 69 to 71, and throughout MS could be omitted as these have proven inaccurate and obsolete descriptions of both pfemp1 geno- and phenotypes. If kept i suggest some subtle changes below:
Line 73: For instance, half of group A and some B variants (DC8) bind …, whereas group A and C variants bind CD36 and are… (ie remove “tend to”).

Answer:

We appreciate the reviewer’s perspective regarding the validity of domain cassettes (DCs). We recognise that there has been debate about their accuracy; however, many recent studies on *var* gene expression and pathogenesis continue to use DC classification as a point of reference. For this reason, we believe it remains useful to include this terminology for readers who may encounter it in the literature.

As suggested, we have revised line 73 to read:

“For instance, group A and some B variants (DC8) bind to EPCR and are associated with cerebral malaria, whereas group B and C variants bind to CD36 and are linked to milder infections “

Similarly the commonly used phrase "such as" on PfEMP1 endothelial receptors should changed as 1) it implies there is something grouping and common about CD36 EPCR and ICAM1 among endothelial receptors- there is not to my knowledge..2) also it implies some unspecific binding to a panel of receptors within ec receptors - this is not the case either..PfEMP1 have shown to be highly adapted to their human receptors.

I suggest "via specific binding to host receptors including cd36, EPCR and ICAm1..."

Answer:

Thank you for highlighting this point and for suggesting clearer wording. We agree that “such as” could be misleading in this context. The introduction has been modified as suggested (lines 50–51) and now reads:

*Pf*EMP1 enables iRBCs to adhere to endothelial cells via specific binding to host receptors including CD36, EPCR, or ICAM1, allowing parasites to avoid splenic clearance but contributing to microvascular obstruction and inflammation, hallmarks of malaria pathology

Line 64: strictly speaking: var2csa is not conserved ....

Answer:

The line has been modified to ‘nearly conserved sequence’ and now reads:

" Group E comprises the unusual var gene named var2csa, which has a nearly conserved sequence.”

Section from line 156: The use of "var" gene sequence regions is misleading as what was used is the translated pfemp1 (protein) sequences.

Answer:

Yes, we agree. We modified it to ‘*Pf*EMP1’ and is now:

“To predict upstream types from *Pf*EMP1 sequences, we tested Support Vector Machines (SVMs), Random Forest (RanFor), and XGBoost (XGB) across four categories of partial protein sequence types...”

Reviewer #2:

The authors address an important issue, the ability to infer further information about var gene sequences based on the partial sequences typically obtained from volunteer samples. This has value for evolutionary and genetics perspectives, attempting to understand the recombinations that underly the phylogeny of these gene family, and also may prove useful in identifying associations between var sequences and clinical phenotypes. Associations between ups types and severe disease have been frequently noted but remain unexplained, a robust means of predicting characteristics of these genes in the absence of the definitive upstream sequence data will allow such associations to be tested in larger datasets. The ultimate goal would be to predict the set of genes to which a tag or contig could belong and this work is a valuable step along that path.

The developed tool has advantages and disadvantages compared to the already available cUPS namely upsAI classifies ups B better and cUPS classifies ups C better, this appears to be because upsAI claims more Cs as Bs and cUPS claims more Bs as Cs. As the authors note this difference affects accuracy due to the greater number of B than C genes in the genome. Combining the two approaches would enable a relatively stringent set of identifications to be made but would leave a large number of genes as either upsC or B.

The authors made the interesting biological observations that he tools worked best on models that segregated genes by their chromosome position rather than by ups B/C. This suggests that constrained recombination due to genomic location has segregated the coding sequences for chromosomal internal upsB despite the homology between upsB sequences from telomeres or internal sites. Their tag analysis further substantiates this showing that tags can be associated with another ups type than the actual gene they belong to, presumably due to recombination.

Overall this paper makes a useful contribution providing a novel tool for var gene classification and using it to make several interesting observations about var gene recombination that inform our understanding of the gene family's evolution.

Answer:

We would like to thank the reviewer for their feedback and positive evaluation. We agree that for tags, Cups and upsML could be combined, however, the advantage of upsML is that it is designed and tested on different input types.

I have only 2 minor suggestions,
Line 22 "the best model of upsAI for DBLα-tags sequences achieves an overall accuracy of 83%, 92% and for full-length PfEMP1 sequences, therefore significantly outperforming existing tools."
There appears to be a typo in this sentence

Answer:

Thank you for spotting this, we modified it to:

"The best model of upsML for DBLα-tags sequences achieves an overall accuracy of 83%, and 92% for full-length PfEMP1 sequences..."

Lines 388-391
"However, we advise caution in interpreting these patterns, as the underlying dataset is subject to biases related to sequencing quality and assembly methods. To draw robust biological conclusions regarding var gene distributions, more controlled datasets, such as RNA-Seq-derived contigs from clinical isolates stratified by disease phenotype, would be necessary. "
Why would rnaseq be more informative about var distributions? Presumably not all the var repertoire would be expressed and there are more likely to issues with uneven 5' coverage from rnaseq. Clinical rnaseq would allow associations between ups type and clinical disease to be identfied. Could the authors clarify what they mean by this statement.

Answer:

Thank you for pointing this out. We agree that our original wording was unclear. Our intention was not to imply that RNA-Seq would provide a complete picture of *var* gene distributions across the entire repertoire. Rather, we meant that RNA-Seq datasets from clinical isolates—especially when processed with consistent protocols and stratified by disease phenotype—could enable associations between *ups* types and clinical outcomes to be explored in a controlled manner.

To clarify, we have revised the text as follows:

"However, we advise caution in interpreting these patterns, as the underlying dataset is subject to biases related to sequencing quality and assembly methods. To draw robust biological conclusions regarding *var* gene distributions, more controlled datasets processed under uniform protocols should be used. For example, large-scale datasets such as Pf7, or RNA-Seq-derived contigs from clinical isolates stratified by disease phenotype, could enable more reliable associations between *ups* types and clinical outcomes to be identified."

Reviewer #3: from attached PDF.

In this paper, the authors develop a new machine learning method (based on support vector machines) to classify the upstream sequences of the var genes of Plasmodium falciparum, based on the protein sequences (or subsequences) of the gene itself. They show that their method is able to successfully classify with high accuracy, including on the upsB/C divide which has previously been difficult to separate, and outperforms current methods in the literature.

The paper is well-written and easy to read, and the problem it tackles is interesting and topical. The results are good, and represent a significant advance on the current state-of-the-art. There are some issues that I would like to see addressed, and areas that I would like to see explored a bit further, but overall I think it is a nice addition to the literature.

Major comments:

1. The characterisation of the method as an "AI" method is inaccurate. The methods used here are well-known and well-established machine learning methods, but they are not AI methods in the modern sense of deep learning/neural networks. Having the term "AI" in the title and in the name of the method is misleading at best; when I read the title, I was indeed expecting some kind of deep neural network. To further characterise the method as "explainable AI", when this is unrelated to the modern

(and topical) sense of extracting meaning from “black box”-style models, seems to me to be a little disingenuous.

Answer:

This is indeed a very valid point, and we renamed the tools throughout the manuscript, GitHub and on the command line.

2. Why were the models only trained with 1-, 2-, and 4-mers? The improving performance with respect to length suggests that longer lengths should have been tried. I understand that at some point there are too many feature dimensions, but certainly 3-mers (at the very least) should have been tried.

Answer:

Thank you for this suggestion. Our initial choice of 1-, 2-, and 4-mers was guided primarily by computational efficiency, as the feature space grows exponentially with increasing *k*-mer length and models with $k \geq 5$ became computationally impractical for our datasets.

In response to the reviewer’s comment, we re-ran the analyses using the fastest model (SVM Linear) with 3-mers included. As expected, performance improved with longer *k*-mers: 3-mers outperformed 2-mers, and 4-mers outperformed 3-mers, although the performance gains between successive *k*-mer lengths were modest (see Table below).

	Tripeptied (k=3)	Tetrapeptied (k=4)
tag	0.8350	0.8350
cassette	0.8421	0.8697
‘exon1’	0.8845	0.8950
PfEMP1	0.9111	0.9190

3. It is somewhat improper to attempt several different machine learning methods and report the best classification accuracy among them as the final numerical answer. Due to random variation, one of the methods will always be slightly better than the others, but not systematically. Indeed, this is exactly what we see in (e.g.) Table 11, where there is (to my eye) no significant difference between the methods, but the best number among them is being claimed as the “overall accuracy”. This leads to different methods being used for different input data types, for no biological reason.

What should be done is that a single method should be chosen based on this Table (probably the linear SVM), and the accuracy of that one method reported from then on.

Answer:

We appreciate the reviewer's point regarding the interpretation of classification accuracy across multiple methods. Our intention in presenting results from several machine learning methods was to provide users with a **suite of approaches** rather than to claim superiority of a single model. While we agree that the observed differences in overall accuracy are small, we included multiple methods to highlight potential trade-offs in **sensitivity and specificity** across different sequence types (e.g., tag sequences, domains, exon 1, and full-length genes).

Because *upsML* allows users to analyse a variety of input data types, we believe it is informative to report the performance of multiple methods so that users can select the most appropriate approach for their specific dataset and research question. For this reason, we would prefer to keep the representation as is while clarifying in the text that the differences in overall accuracy are modest and not the main focus of our comparisons.

4. l289: I would not characterise this as a "classifier for genome location"; I would expect such a thing to predict the actual position in the genome. What is being done here is rearranging the upsB/C divide according to genome location. I think this is interesting enough in its own right without overselling it. Likewise, I would consider l325–326 as somewhat overselling the ability to predict chromosomal position.

Answer:

We appreciate the reviewer's point regarding the terminology used for this analysis. Our goal was not to imply prediction of absolute chromosomal coordinates but rather to classify *var* genes into the well-established **subtelomeric versus internal** genomic locations. Since the *Plasmodium falciparum* genome was first published in 2003, these two locations have been recognised as biologically meaningful, with recombination rates and phenotypic associations differing by location.

Given the difficulty in distinguishing *upsB* and *upsC* groups, we felt it was important to highlight this classification explicitly, as it provides a novel way to integrate chromosomal positioning into *var* gene analyses. To clarify this point and avoid any sense of overstatement, we have revised the discussion as follows:

“Given the difficulty in distinguishing *upsB* and *upsC*, and the interest in chromosomal positioning of *var* genes, we developed an additional classifier to distinguish subtelomeric from internal *var* genes (Table 5). ≈ .”

5. Figure 4: In general I like this idea a lot and I think it clearly shows the inclination of various subsequences towards the different ups types, which to my knowledge has not been studied before. However, the details of the method used here need to be expanded; the Methods section (l539) does not give the mathematical formulas used. I can guess what is going on, but I should not have to. In the Results section, the AUC score is not defined in l339. I was hoping that it would be defined later, but when I read the relevant Methods section, there does not appear to be any mention of AUC.
In fact, I think more could be done in this direction. The particular sequence studied shows a tendency towards upsB in the tag region but upsC in other parts of the PfEMP1 sequence. In the discussion this is considered as a signal of recombination. There is already a well-known method (Zilversmit et al., cited in the paper) for detecting recombination in var genes; why not use it here to determine that there has indeed been a recombination in this particular sequence, and what the parents were? That would lend considerably more weight to this analysis.
When a linear projection is used for classification, the “loading” weights (the coefficients of the projection matrix) are usually used to explain the relative importance of the various features. In this case, this seems like an obvious thing to do, and would ascribe importance to the various 4-mers which could be then investigated for biological meaning. Was this considered?
Finally, the claim (l419) that the drops are aligned with recombination breakpoints should be substantiated, by (e.g.) comparison to homology block (HB) locations.

First, we agree that we have to include the calculation of the AUC into the methods, which we did. The calculation of feature importance is explained under “Creating and Testing Machine Learning Models”:

“Feature importances were estimated for Tetrapeptide linear SVM model by fitting models that classify var sequences into the two classes var group B/C or internal/external *var* genes.

We used the weights of the SVM models, equivalent to the coordinates of the vector orthogonal to the hyperplane separating the two classes, as direct measures of each feature (amino acid sequence) tendency to classify a *var* sequence into each class. Using a sliding window, we then mapped the relative feature importances of each tetrapeptide along full Pfemp1 sequences to identify regions of Pfemp1 sequences associated with different classes."

And later the calculation of AUC as used in Figure 4 is now covered in "Explainable AI":

"The area under the curve (AUC) represents the cumulative sum of Regional Decision Weight contributions along the sequence. These AUC values illustrate how tetrapeptide features progressively influence the classification outcome from the start to the end of the sequence. A full-gene AUC (red) was computed across the entire sequence to capture the overall classification bias, whereas a subregional AUC (green) was restricted to a defined interval (e.g., amino acids 180–300) to quantify the local contribution of features within that region."

We do agree that these findings are interesting and will indeed follow them up in the lab. However, this paper proposes a new method to classify PfEMP1 we feel that the reviewers comment would be out of scope. It would change the paper to a more evolutionary nature.

6. l379: There should be more analysis of the results here than a simple table. The text considers that it is "informative to observe geographical variation", but what information should be observed here?

Answer:

Again, similar to the previous comment, we feel that analysis more those results would change the scope of the paper. Important here was to point out, that it is feasible to do. However, we added a bit more details and an outlook, which reads like:

However, we advise caution in interpreting these patterns, as the underlying dataset is subject to biases related to sequencing quality and assembly methods. To draw robust biological conclusions regarding *var* gene distributions, more controlled datasets processed under uniform protocols should be used. For example, large-scale datasets such as Pf7, or RNA-Seq-derived contigs from clinical isolates stratified by disease phenotype , could enable more reliable associations between *ups* types and clinical outcomes to be identified.

7. l506: I am somewhat disturbed that using 1-mers can achieve any classification value at all. This is equivalent to using (single) amino acid frequencies to classify the se-

quences, analogous to using GC-content for DNA sequences. There should be very 2little discriminatory value there, but instead the performance (for e.g., tags) is only slightly worse than the full method. Why is this so?

More generally, the method does not use any (sequence) positional information other than local (sub-)sequences in the form of k-mers. Again, why is this? Is position unnecessary for classification?

Answer:

We appreciate the reviewer's observation and agree that it is somewhat surprising that the 1-mer representation already provides meaningful classification power. Similar findings have been reported in other studies, such as Kumar *et al. doi:10.1186/1471-2105-8-463* [REF], where amino acid composition alone was sufficient for discriminating between protein classes. In our case, the result suggests that even simple sequence properties—such as overall nucleotide or amino acid composition (e.g., GC content)—carry biological signal relevant to *ups* type classification.

As noted, our current approach does not incorporate positional information and relies solely on *k*-mer frequencies. We agree that methods leveraging positional context, such as recurrent neural networks or other deep learning models, could offer additional insights once larger training datasets become available. We have added the following clarification to the manuscript:

"Overall, larger k-mers generated better results and the SVM models were slightly better than the other methods. Interestingly, the SVM model using only 1-mers already provides informative classification performance, suggesting that simple sequence composition features (e.g., GC content) carry signal for *ups* type discrimination. Future work using larger datasets could explore methods that incorporate positional information, such as recurrent neural networks or other deep learning approaches."

8. There is somewhat of an overall lack of mathematical detail in the description of the methods. I am reasonably familiar with the methods used, but the manuscript should be self-contained. Also, it appears that the methods were used "out-of-the-box" without specific adaptation to the task at hand. This is okay, but perhaps not ideal.

Answer:

We appreciate the reviewer's concern regarding the level of mathematical detail in the methods. Our approach primarily used established machine learning algorithms with standard implementations, so we felt that citing the primary references and clearly describing our parameter settings would allow the manuscript to remain self-contained without reproducing well-known mathematical derivations.

In response to this comment, we have expanded the methods section to clarify how key metrics such as the AUC were computed in our context. Specifically, we now describe how the area under the curve (AUC) represents the cumulative contribution of regional decision weights along the sequence:

"The area under the curve (AUC) represents the cumulative sum of regional decision weight contributions along the sequence. A full-gene AUC was computed across the entire sequence to capture the overall classification bias, whereas a subregional AUC was restricted to a defined interval (e.g., amino acids 180–300) to quantify the local contribution of features within that region."

We believe this additional detail strikes a balance between transparency and readability, while ensuring the manuscript remains focused on the biological application rather than on re-deriving established machine learning concepts.

Minor comments:

Answer:

We would like to thank the reviewer for flagging up those issues. We have addressed them

• In general, there should be a space before citations, e.g., l51: "to the iRBC surface

(1)".

We presume the editing services will use the PlosOne format for References?

• l22: "and 92% for"

Modified.

• l75: "var2csa" (capitalisation)

The full capitalisation is used when referring to the protein, which is the case here.

• l84: Refer to Figure 1 in the text.

Added now.

• l85: I suggest using parentheses, e.g., “subtelomeric regions (upsA/B/E)” as the current punctuation is unclear.

Modified.

• l138: This seems to imply that 110 out of the 172 genes were identified as upsE, which seems absurd — I suggest rewording.

Modified. 110 was indeed 5.

• Figure 2: To better understand the reliability of the annotations, the annotations mapped from the Rask dataset should be highlighted in this figure, or better yet shown by themselves in a companion figure.

Answer:

We discussed this, however, we just label a branch if there is evidence. If there is no Rask evidence, there is no association with UPS types. Also, adding the information would make the tree less readable.

• l162: To exclude a particular genome for validation risks that the genes included there are not a random sample; I think this is probably okay in this instance since this genome is not used to assess accuracy.

We thought that this would be the best form to test this here.

• l167: “with tetrapeptides, and we did”

Corrected

- l185: incomplete sentence

Corrected (l184)

- Table 4: extra M in “HMM”

Corrected

- l252: last sentence is ungrammatical

Modified
