# Supplementary material for: upsML: A high-accuracy machine learning classifier for predicting Plasmodium falciparum var gene upstream groups
Source: PLoS One. 2026 Apr 16;21(4):e0344557. doi: 10.1371/journal.pone.0344557 (PMC13086428; doi:10.1371/journal.pone.0344557)
Supplement: Attachment — Submitted filename: plosone25b.pdf [file pone.0344557.s011.pdf]

Review of "upsAI: A high-accuracy machine learning classifier for predicting *Plasmodium falciparum var* gene upstream groups"

In this paper, the authors develop a new machine learning method (based on support vector machines) to classify the upstream sequences of the *var* genes of *Plasmodium falciparum*, based on the protein sequences (or subsequences) of the gene itself. They show that their method is able to successfully classify with high accuracy, including on the upsB/C divide which has previously been difficult to separate, and outperforms current methods in the literature.

Although the authors have made a credible attempt to address my points, and have mostly done so successfully, there remain some points which I am not completely convinced about. I go through these below, including my original review and the authors' responses in italics.

Major comments:

1. It is somewhat improper to attempt several different machine learning methods and report the best classification accuracy among them as the final numerical answer. Due to random variation, one of the methods will always be slightly better than the others, but not systematically. Indeed, this is exactly what we see in (e.g.) Table 1, where there is (to my eye) no significant difference between the methods, but the best number among them is being claimed as the "overall accuracy". This leads to different methods being used for different input data types, for no biological reason. What should be done is that a single method should be chosen based on this Table (probably the linear SVM), and the accuracy of that one method reported from then on.

   We appreciate the reviewer's point regarding the interpretation of classification accuracy across multiple methods. Our intention in presenting results from several machine learning methods was to provide users with a suite of approaches rather than to claim superiority of a single model. While we agree that the observed differences in overall accuracy are small, we included multiple methods to highlight potential trade-offs in sensitivity and specificity across different sequence types (e.g., tag sequences, domains, exon 1, and full-length genes).

Because upsML allows users to analyse a variety of input data types, we believe it is informative to report the performance of multiple methods so that users can select the most appropriate approach for their specific dataset and research question. For this reason, we would prefer to keep the representation as is while clarifying in the text that the differences in overall accuracy are modest and not the main focus of our comparisons.

I'm afraid I am not really convinced by this argument. The problem here is that there is no biological distinction between the various approaches; rather the distinction is statistical, through the use of different kernel functions. If there were credible reasons that one of these kernel functions would work well for a certain data type and a different one would work well for another type, then it would make sense to present multiple approaches. However, as I observed earlier, the variation between the methods appears to be largely random noise, so that different approaches work better for different types simply because there must be a best method. Biologically, there is no reason that one should use e.g., a polynomial kernel for tags but a linear kernel for cassettes. From an end-user perspective, the approaches are largely interchangeable, and it is difficult to resolve conflicting outcomes. I think one should not resolve these differences based on differences in classification accuracy of 0.01.

2. Figure 4: In general I like this idea a lot and I think it clearly shows the inclination of various subsequences towards the different ups types, which to my knowledge has not been studied before. However, the details of the method used here need to be expanded; the Methods section (l539) does not give the mathematical formulas used. I can guess what is going on, but I should not have to. In the Results section, the AUC score is not defined in l339. I was hoping that it would be defined later, but when I read the relevant Methods section, there does not appear to be any mention of AUC.

   First, we agree that we have to include the calculation of the AUC into the methods, which we did. The calculation of feature importance is explained under "Creating and Testing Machine Learning Models": (text removed for brevity)

   And later the calculation of AUC as used in Figure 4 is now covered in "Explainable AI": (text removed for brevity)

   The Methods section is still not detailed enough for me to fully understand what is going on. (For context, I am a mathematician, and I would like to know the mathematical details used here.) There is a repeated reference to the 'weight' of a tetrapeptide — how is that defined and extracted from the SVM model? The "Regional Decision Weight" is not clearly defined — for example, how large are the windows used? I also question if it makes sense to sum Regional Decision Weights along the sequence, since they are already local aggregates in a window. Similarly, what curve is the AUC the area under of? The traditional formulation of AUC as the area under an ROC or sensitivity/specificity curve doesn't seem to apply here. What I would really like to see are concrete mathematical formulas that enable the reader to exactly identify and

reproduced what is being calculated, and I think the authors have not gone far enough in this respect.

3. l379: There should be more analysis of the results here than a simple table. The text considers that it is "informative to observe geographical variation", but what information should be observed here?

   Again, similar to the previous comment, we feel that analysis more those results would change the scope of the paper. Important here was to point out, that it is feasible to do.

   I do not think that a large amount of extra analysis is needed here; all I am asking for is for the authors to interpret the numbers given in the table. Since this is apparently feasible, I would suggest that it be done.

Minor comments:

- l140: "mapping of all of them"
- Table 3: The table is now too wide for the page.
- Table 4: The table now appears to be duplicated, and the first part of the second table looks a bit weird.
- Table 5: The caption "Internal/telomeric" is not really in a sensible place.
- l398: Doubled sentence
- l421: Although I take the meaning, GC content is not a property of amino acid composition.
- l532: "the tetrapeptide linear SVM model"
- l562: Since all other instances of "AI" have been removed from the text, this heading no longer seems appropriate. Likewise the keyword (l47).
- l592: "mentioned in the Methods".
- l598: Heading formatting
