# Supplementary material for: upsML: A high-accuracy machine learning classifier for predicting Plasmodium falciparum var gene upstream groups
Source: PLoS One. 2026 Apr 16;21(4):e0344557. doi: 10.1371/journal.pone.0344557 (PMC13086428; doi:10.1371/journal.pone.0344557)
Supplement: Attachment — Submitted filename: plosone25c.pdf [file pone.0344557.s013.pdf]

Review of "upsAI: A high-accuracy machine learning classifier for predicting *Plasmodium falciparum var* gene upstream groups"

In this paper, the authors develop a new machine learning method (based on support vector machines) to classify the upstream sequences of the *var* genes of *Plasmodium falciparum*, based on the protein sequences (or subsequences) of the gene itself. They show that their method is able to successfully classify with high accuracy, including on the upsB/C divide which has previously been difficult to separate, and outperforms current methods in the literature.

The authors have responded well to the points raised in my latest review. With one exception, I am happy with the changes and any remaining comments are minor.

Major comment (original comment and response first):

1. The Methods section is still not detailed enough for me to fully understand what is going on. (For context, I am a mathematician, and I would like to know the mathematical details used here.) There is a repeated reference to the 'weight' of a tetrapeptide — how is that defined and extracted from the SVM model? The "Regional Decision Weight" is not clearly defined — for example, how large are the windows used? I also question if it makes sense to sum Regional Decision Weights along the sequence, since they are already local aggregates in a window. Similarly, what curve is the AUC the area under of? The traditional formulation of AUC as the area under an ROC or sensitivity/specificity curve doesn't seem to apply here. What I would really like to see are concrete mathematical formulas that enable the reader to exactly identify and reproduced what is being calculated, and I think the authors have not gone far enough in this respect.

   We now understand the detail the referee wants to see. From line 581, we described in more details what was done. Especially, we define the "weights" and the windows size. We chose to describe this as the code we used, as it would make it easier for the readership than to read mathematical descriptions. We hope that the editor and reviewer agree here.

   While I appreciate the effort the authors have made to address this point, and I do find it sufficiently detailed now, I unfortunately do not agree that it is better explained via

code. While mathematics is a universal language (to a point), Python is not, and it is not beyond imagining that there are potential readers of the paper who do not know it. Using code makes the definitions not self-contained, since many of the variables are not previously defined (presumably being defined somewhere else in their code), and neither are the functions, so that the reader must either know what they do already or look them up somewhere else, neither of which is a desirable situation. It also makes things harder to read since there are also many lines which are unnecessary for understanding (e.g., "sequence = translate_sequence(sequence)" or "from sklearn.svm import LinearSVC"). In my opinion, self-contained pseudocode is acceptable, but straight code is not.

Now that I do have an idea what is going on here I can comment a bit on the method itself. I think it is largely sensible, however some of the names are poorly chosen, which contributed to my confusion in earlier versions which were not as fully explained. The "regional decision weight" does not appear to be a "regional" weight at all, but a positional weight which is then summed over a region to create a regional weight. Likewise, the "area under the curve" does not appear to be the area under any curve (and certainly does not correspond to the traditional definition of AUC), but a cumulative sum of the positional weights. I think both these concepts are valid, but would suggest that they be renamed.

Minor comments:

- l32: space before "[17]"
- l142: "mapping of all of them", not "mapping all of them"
- l197: Since the method with the lowest runtime is always the linear SVM, I do not see why the authors cannot just say that they recommend the linear SVM.
- l283: opening double-quotes for " "Known" "
- l309: "subtelomeric"
- l384: space before "(average of 48)"
- l396: Specify the statistical test used here.
- l398: It is interesting, and perhaps worth mentioning, that although the differences in Table 6 (bottom) are less significant, there is still a clear separation between the regions, with East African countries almost all on the bottom and all Asian countries on the top. I wonder if a rank-sum test could be used here.
- l467: "*var* gene biology"
- l470: space before "[4]"

- l481–3: “More broadly” is used in two sentences in a row, which sounds awkward.
- l492: capitalise “Python”
- l546: “*var* gene sequences”
- l583: “two classes, *var* group B/C or...”
- l592: “mentioned in the Methods”, not “mentioned in the method”
