# Supplementary material for: upsML: A high-accuracy machine learning classifier for predicting Plasmodium falciparum var gene upstream groups
Source: PLoS One. 2026 Apr 16;21(4):e0344557. doi: 10.1371/journal.pone.0344557 (PMC13086428; doi:10.1371/journal.pone.0344557)
Supplement: Attachment — Submitted filename: UpsML.PlosOne.Letter_rebuttal2.docx [file pone.0344557.s012.docx]

Dear Editor,

Please find our rebuttal letter. We have actually found interesting insight (point 3) and have added more information about the Explainable AI.

We are confident that we addressed the issues raised by the reviewer.

Best wishes,

Thomas Otto

Reviewer 3:

In this paper, the authors develop a new machine learning method (based on support

vector machines) to classify the upstream sequences of the var genes of Plasmodium falci-

parum, based on the protein sequences (or subsequences) of the gene itself. They show that

their method is able to successfully classify with high accuracy, including on the upsB/C

divide which has previously been difficult to separate, and outperforms current methods in

the literature.

Although the authors have made a credible attempt to address my points, and have

mostly done so successfully, there remain some points which I am not completely convinced

about. I go through these below, including my original review and the authors’ responses in

italics.

Major comments:

1. It is somewhat improper to attempt several different machine learning methods and

report the best classification accuracy among them as the final numerical answer.

Due to random variation, one of the methods will always be slightly better than the

others, but not systematically. Indeed, this is exactly what we see in (e.g.) Table

1, where there is (to my eye) no significant difference between the methods, but the

best number among them is being claimed as the “overall accuracy”. This leads to

different methods being used for different input data types, for no biological reason.

What should be done is that a single method should be chosen based on this Table

(probably the linear SVM), and the accuracy of that one method reported from then

on.

*We appreciate the reviewer’s point regarding the interpretation of classification accu-*

*racy across multiple methods. Our intention in presenting results from several machine*

*learning methods was to provide users with a suite of approaches rather than to claim*

*superiority of a single model. While we agree that the observed differences in overall*

*accuracy are small, we included multiple methods to highlight potential trade-offs in*

*sensitivity and specificity across different sequence types (e.g., tag sequences, domains,*

*exon 1, and full-length genes).*

*Because upsML allows users to analyse a variety of input data types, we believe it*

*is informative to report the performance of multiple methods so that users can select*

*the most appropriate approach for their specific dataset and research question. For*

*this reason, we would prefer to keep the representation as is while clarifying in the*

*text that the differences in overall accuracy are modest and not the main focus of our*

*comparisons.*

I’m afraid I am not really convinced by this argument. The problem here is that there

is no biological distinction between the various approaches; rather the distinction is

statistical, through the use of different kernel functions. If there were credible reasons

that one of these kernel functions would work well for a certain data type and a different

one would work well for another type, then it would make sense to present multiple

approaches. However, as I observed earlier, the variation between the methods appears

to be largely random noise, so that different approaches work better for different types

simply because there must be a best method. Biologically, there is no reason that one

should use e.g., a polynomial kernel for tags but a linear kernel for cassettes. From an

end-user perspective, the approaches are largely interchangeable, and it is difficult to

resolve conflicting outcomes. I think one should not resolve these differences based on

differences in classification accuracy of 0.01.

**Response:**

We agree that we might have been focusing on small differences here. However, we do think it is legitimate to test different methods and understand sensitivity and specificity. Hence, we change the message of the paper as requested.

For example in the abstract we write:
Several models in upsML achieve an accuracy of 83% for DBLα-tags sequences and 92% for full-length *Pf*EMP1 sequences. – rather than the best method.

Next in the results we wrote:

The differences between models within each sequence type were small (1–2%), **which were not statistically significant**.

Several models perform similarly; therefore, we recommend using the one with the lowest runtime.

We do think that it is interesting for the reader to under the differences between the models for the false positive (Table 2) and Fig 3 – the Pf3D7 test.

And finally, we already wrote:

However, for tag sequences, some models offered marginally higher accuracy at significant computational cost - up to 100-fold slower than others. Given this, we opted for the fastest-performing model (linear SVM) for most input types and the polynomial SVM for tags. With trained models, annotating 1,200 *var* genes from 20 genomes takes approximately one second using linear SVM and 100 seconds using the polynomial model

So we think that with the changes, we make it clear that the approaches are very similar and we suggest to use the one that has a shorter runtime.

Comment 2.

Figure 4: In general I like this idea a lot and I think it clearly shows the inclination

of various subsequences towards the different ups types, which to my knowledge has

not been studied before. However, the details of the method used here need to be

expanded; the Methods section (l539) does not give the mathematical formulas used. I

can guess what is going on, but I should not have to. In the Results section, the AUC

score is not defined in l339. I was hoping that it would be defined later, but when I

read the relevant Methods section, there does not appear to be any mention of AUC.

*First, we agree that we have to include the calculation of the AUC into the methods,*

*which we did. The calculation of feature importance is explained under “Creating and*

*Testing Machine Learning Models”: (text removed for brevity)*

*And later the calculation of AUC as used in Figure 4 is now covered in “Explainable*

*AI”: (text removed for brevity)*

The Methods section is still not detailed enough for me to fully understand what is

going on. (For context, I am a mathematician, and I would like to know the mathemat-

ical details used here.) There is a repeated reference to the ‘weight’ of a tetrapeptide

— how is that defined and extracted from the SVM model? The “Regional Decision

Weight” is not clearly defined — for example, how large are the windows used? I also

question if it makes sense to sum Regional Decision Weights along the sequence, since

they are already local aggregates in a window. Similarly, what curve is the AUC the

area under of? The traditional formulation of AUC as the area under an ROC or

sensitivity/specificity curve doesn’t seem to apply here. What I would really like to

see are concrete mathematical formulas that enable the reader to exactly identify and

reproduced what is being calculated, and I think the authors have not gone far enough

in this respect.

**ANSWER:**

We now understand the detail the referee wants to see. From line 581, we described in more details what was done. Especially, we define the “weights” and the windows size. We chose to describe this as the code we used, as it would make it easier for the readership than to read mathematical descriptions. We hope that the editor and reviewer agree here.

Comment 3. l379: There should be more analysis of the results here than a simple table. The

text considers that it is “informative to observe geographical variation”, but what

information should be observed here?

ANSWER:

We wrote know down the interesting information, that the relative frequency of internal var genes is high in Asia.

We added the text in three part of the paper.

**Abstract**

*Additionally, we developed a model to distinguish internal from subtelomeric var genes, which we applied to a global collection of P. falciparum genomes, revealing a higher frequency of internal var genes in Asia.*

**Results**

*In contrast, our positional classifier revealed a consistent enrichment of internal CB-type var genes in isolates from Asia regions (Table 6, top). Frequencies were highest in Cambodia and Thailand (33.9% each) and lowest in Kenya and Mali (26.7% and 26.8%, respectively). To better understand those differences in all samples, we grouped the countries into East Africa (Kenya, Malawi, Congo – median 28.1%), West Africa (Senegal, The Gambia, Ghana, Mali – median 28.8%), and Asia (Thailand, Cambodia, Vietnam, Laos – median 32.6 %, Figure S1). Differences in internal var gene frequency were significant between Asia and the two African regions, p-values 3.4e-12 (West Africa) and 2.1e-09 (East Africa), but not within Africa (p-value = 0.04). In contrast, we did not observe a similarly clear difference for upsC (Table 6, bottom), likely reflecting the known difficulty of distinguishing upsB from upsC based on sequence features alone. Thus, internal var genes are significantly more frequent in Asia.*

**Discussion**

*Applying this positional classifier to a larger global var gene dataset [20] (Table 6), we observed that the relative abundance of internally located var genes varies geographically. A major contrast between Asian and African parasite populations is transmission intensity, with higher transmission in central and eastern Africa and lower transmission in Asia. In low-transmission areas, parasites are expected to persist longer within individual hosts, favouring chronic infections. Nobel and colleagues [25] predicted that chronicity would be associated with increased expression of upsC (internal) var genes, and our analysis supports this prediction. On the other hand, because those numbers are relative, isolates in Africa may have more subtelomeric var genes, perhaps due to different clinical phenotypes or multiple infections, to maintain higher diversity. More broadly, we note that upsML can be used to generate novel hypotheses and help interpret epidemiological differences in var gene architecture.*

Minor comments:

Thank you for spotting those issues in the text.

• l140: “mapping of all of them”

We are not sure what the reviewer means here:

Line 139 - 141 Sequences annotated as upsE, corresponding to the

conserved *var2csa*, were excluded from phylogenetic-based clustering due to their distinct and

well-characterised nature.

• Table 3: The table is now too wide for the page.

Fixed, but I hope that the Plos One type setter will also have a look here.

• Table 4: The table now appears to be duplicated, and the first part of the second table

looks a bit weird.

Fixed

• Table 5: The caption “Internal/telomeric” is not really in a sensible place.

Fixed

• l398: Doubled sentence

Fixed.

• l421: Although I take the meaning, GC content is not a property of amino acid com-

position.

Yes, stupid. Now reads:
simple amino acid sequence composition features carry signal

• l532: “the tetrapeptide linear SVM model”

fixed

• l562: Since all other instances of “AI” have been removed from the text, this heading

no longer seems appropriate. Likewise the keyword (l47).

Although we agree that the classifiers in *upsML* are not “AI” in the broad sense, the interpretability analyses we apply do fall within established definitions of Explainable AI (XAI). Our use of linear SVM coefficients for feature importance, the derivation of local and global explanatory profiles, and the regional model diagnostics all correspond to standard XAI methods. For this reason, we feel the heading remains appropriate, and we have clarified these steps in the revised Methods.

• l592: “mentioned in the Methods”.

We agree, but I think we need to keep it here explicitly as format of Plos one.

• l598: Heading formatting

Fixed
