# Supplementary material for: upsML: A high-accuracy machine learning classifier for predicting Plasmodium falciparum var gene upstream groups
Source: PLoS One. 2026 Apr 16;21(4):e0344557. doi: 10.1371/journal.pone.0344557 (PMC13086428; doi:10.1371/journal.pone.0344557)
Supplement: Attachment — Submitted filename: UpsML.RebuttalLetter.V3.docx [file pone.0344557.s014.docx]

Reviewer 3

In this paper, the authors develop a new machine learning method (based on support

vector machines) to classify the upstream sequences of the var genes of Plasmodium falci-

parum, based on the protein sequences (or subsequences) of the gene itself. They show that

their method is able to successfully classify with high accuracy, including on the upsB/C

divide which has previously been difficult to separate, and outperforms current methods in

the literature.

The authors have responded well to the points raised in my latest review. With one

exception, I am happy with the changes and any remaining comments are minor.

Major comment (original comment and response first):

1. The Methods section is still not detailed enough for me to fully understand what is

going on. (For context, I am a mathematician, and I would like to know the mathemat-

ical details used here.) There is a repeated reference to the ‘weight’ of a tetrapeptide

— how is that defined and extracted from the SVM model? The “Regional Decision

Weight” is not clearly defined — for example, how large are the windows used? I also

question if it makes sense to sum Regional Decision Weights along the sequence, since

they are already local aggregates in a window. Similarly, what curve is the AUC the

area under of? The traditional formulation of AUC as the area under an ROC or

sensitivity/specificity curve doesn’t seem to apply here. What I would really like to

see are concrete mathematical formulas that enable the reader to exactly identify and

reproduced what is being calculated, and I think the authors have not gone far enough

in this respect.

**Answer:**

We now understand more clearly the level of methodological detail the reviewer was seeking. In the revised manuscript (from line 581 onward), we expanded the description of the method. In particular, we define the tetrapeptide “weights” and the windows sizes used.

While I appreciate the effort the authors have made to address this point, and I do find

it sufficiently detailed now, I unfortunately do not agree that it is better explained via

1code. While mathematics is a universal language (to a point), Python is not, and it is

not beyond imagining that there are potential readers of the paper who do not know

it. Using code makes the definitions not self-contained, since many of the variables are

not previously defined (presumably being defined somewhere else in their code), and

neither are the functions, so that the reader must either know what they do already

or look them up somewhere else, neither of which is a desirable situation. It also

makes things harder to read since there are also many lines which are unnecessary for

understanding (e.g., “sequence = translate sequence(sequence)” or “from sklearn.svm

import LinearSVC”). In my opinion, self-contained pseudocode is acceptable, but

straight code is not.

**Answer:**

Our initial intention was to describe these steps using the actual implementation logic, as we felt this would be more accessible to the broad PLOS One readership than formal mathematical notation. However, we acknowledge the reviewer’s concern that including raw Python code within the manuscript is not ideal, as it is not self-contained and may hinder readability.

We therefore fully agree with the reviewer on this point and have replaced the Python code with self-contained pseudo-code, with clearly defined inputs, outputs, and intermediate variables. This presentation removes implementation-specific details while preserving the precise computational logic, and we believe it substantially improves clarity and accessibility for readers from different backgrounds.

While we respectfully maintain that formal mathematical expressions are not strictly necessary for this journal and audience, we believe that the revised pseudo-code, combined with the expanded textual explanation, now provides a sufficiently precise and transparent description of the method.

The full, executable implementation remains available on GitHub for readers who wish to explore or reproduce the analysis in detail.

[Reviewer:]

that I do have an idea what is going on here I can comment a bit on the method

itself. I think it is largely sensible, however some of the names are poorly chosen, which

contributed to my confusion in earlier versions which were not as fully explained. The

“regional decision weight” does not appear to be a “regional” weight at all, but a posi-

tional weight which is then summed over a region to create a regional weight. Likewise,

the “area under the curve” does not appear to be the area under any curve (and cer-

tainly does not correspond to the traditional definition of AUC), but a cumulative sum

of the positional weights. I think both these concepts are valid, but would suggest that

they be renamed.

**Answer**:

We agree that some of the original names were potentially misleading and may have contributed to earlier confusion. In response, we have renamed the term “area under the curve” with “Cumulative Score” (CS). The term "regional decision weight” was replaced by “positional weight”.

Minor comments:

• l132: space before “[17]”

Done

• l142: “mapping of all of them”, not “mapping all of them”

Done

• l197: Since the method with the lowest runtime is always the linear SVM, I do not see

why the authors cannot just say that they recommend the linear SVM.

Good point, done

• l283: opening double-quotes for “ “Known” ”

Fixed opening and closing double-quotes - “Known”

• l309: “subtelomeric”

Fixed

• l384: space before “(average of 48)”

Added

• l396: Specify the statistical test used here.

Added that those were Welch Two Sample t-tests.

• l398: It is interesting, and perhaps worth mentioning, that although the differences

in Table 6 (bottom) are less significant, there is still a clear separation between the

regions, with East African countries almost all on the bottom and all Asian countries

on the top. I wonder if a rank-sum test could be used here.

As the focus is on the tool, we prefer to keep it as with the focus on the top table.

• l467: “var gene biology”

Split gene & biology

• l470: space before “[4]”

Space added

2• l481–3: “More broadly” is used in two sentences in a row, which sounds awkward.

Changed the second to “More generally”

• l492: capitalise “Python”

We capitalised all occurences

• l546: “var gene sequences”

Fixed to singular

• l583: “two classes, var group B/C or...”

Added the comma

• l592: “mentioned in the Methods”, not “mentioned in the method”

Done
